# Genetics in the ADHD Clinic: How Can Genetic Testing Support the Current Clinical Practice?

**DOI:** 10.3389/fpsyg.2022.751041

**Published:** 2022-03-08

**Authors:** Lívia Balogh, Attila J. Pulay, János M. Réthelyi

**Affiliations:** Department of Psychiatry and Psychotherapy, Semmelweis University, Budapest, Hungary

**Keywords:** attention deficit hyperactivity disorder (ADHD), genetics, polygenic risk score (PRS), neurodevelopment, endophenotype, comorbidity, psychiatry

## Abstract

Attention-deficit/hyperactivity disorder (ADHD) is a neurodevelopmental disorder with a childhood prevalence of 5%. In about two-thirds of the cases, ADHD symptoms persist into adulthood and often cause significant functional impairment. Based on the results of family and twin studies, the estimated heritability of ADHD approximates 80%, suggests a significant genetic component in the etiological background of the disorder; however, the potential genetic effects on disease risk, symptom severity, and persistence are unclear. This article provides a brief review of the genome-wide and candidate gene association studies with a focus on the clinical aspects, summarizing findings of ADHD disease risk, ADHD core symptoms as dimensional traits, and other traits frequently associated with ADHD, which may contribute to the susceptibility to other comorbid psychiatric disorders. Furthermore, neuropsychological impairment and measures from neuroimaging and electrophysiological paradigms, emerging as potential biomarkers, also provide a prominent target for molecular genetic studies, since they lie in the pathway from genes to behavior; therefore, they can contribute to the understanding of the underlying neurobiological mechanisms and the interindividual heterogeneity of clinical symptoms. Beyond the aforementioned aspects, throughout the review, we also give a brief summary of the genetic results, including polygenic risk scores that can potentially predict individual response to different treatment options and may offer a possibility for personalized treatment for the therapy of ADHD in the future.

## Introduction

Due to its high heritability and neurodevelopmental nature, Attention-Deficit Hyperactivity Disorder (ADHD) is a condition receiving significant interest in psychiatric genetics. The childhood prevalence is 5–7% (Polanczyk et al., 2007), and in one-third of patients, symptoms persist into adulthood (Franke et al., 2018), causing functional impairment of everyday life. Due to the dimensional feature of the core symptoms – inattention, hyperactivity, and impulsivity– and somewhat subjective approach to determine the extent of functional impairment, mapping of possible genetic risk factors for ADHD remains important in both clinical and population samples. In this review, we present the selected literature to give a summary of the current knowledge about the genetic perspective of ADHD, which highlights the gaps that emerge from the approaches taken by candidate gene and genome-wide association studies (GWAS). Besides the aforementioned aspects, we also present the relevant results of the polygenic risk score (PRS) approach (Torkamani et al., 2018; Martin et al., 2019; Ronald et al., 2021), which can individually estimate the genetic liability for the disorder and ADHD-related traits, thereby offering further insight into the background of the symptomatic and genetic heterogeneity characterizing ADHD. Despite the many limitations to its application, it might hold the promise of creating a bridge between research and the clinic, from ‘bench to bedside.’

### Attention-Deficit/Hyperactivity Disorder Genetic Risk: Heritability and Epigenetic Effects

The heritability of ADHD is high compared to most psychiatric disorders and compares to the heritability rates found in autism spectrum disorder (ASD), bipolar disorder, and schizophrenia (Sullivan et al., 2012). However, our knowledge of the underlying genetic architecture of ADHD remains limited. The familial aggregation of the disorder is strong, and current results suggest that the relative risk of ADHD is 5- to 10-fold for first-degree relatives (Biederman et al., 1990; Biederman, 2005; Franke et al., 2012), which is applicable to core symptoms and is gender-independent (Taylor et al., 2016; Martin et al., 2018). However, regarding the exact characterization of heritability, our results are inconsistent. Based on family and twin studies in recent decades, the heritability of ADHD has been estimated at 77–88% (Faraone and Larsson, 2019). Surprisingly, a mega-analysis of the results from existing GWAS found that the proportion of heritability based on single nucleotide polymorphisms (SNPs) is only 22% (Demontis et al., 2019). Thus, the gap between the results originating from family or twin studies and SNP heritability based on GWAS is huge, a phenomenon commonly referred to as ‘hidden or lost heritability.’ There are several potential explanations for the inconsistent results, of which the methodological differences and the currently incomplete knowledge of the genetic architecture underlying ADHD receive the most attention.

One explanation is that the overall effect of the SNPs that impact ADHD is relatively small. Moreover, the allele frequency of rare variants is below the detection threshold, because linkage disequilibrium (LD) is low; therefore, the GWAS do not have enough statistical power to detect them (Hong and Park, 2012; Visscher et al., 2017). Even in the mega-analysis summarizing the currently available GWAS results, there were only 12 significant hits in a sample over 20,000 cases (Demontis et al., 2019). Therefore, rare variants (having an allele frequency lower than 0.05) that are potentially related to ADHD, such as copy number variants (CNVs), which are genomic segments ranging from 1 Kb to several Mb in the DNA occurring as multiple copies or deletions of a certain chromosomal section, and single nucleotide variants (SNVs) have become the focus of research. The role of CNVs in both childhood (Williams et al., 2010; Stergiakouli et al., 2012; Martin et al., 2015) and adult ADHD (Ramos-Quiroga et al., 2014) has been found to be significant in several studies, and a significant overlap with the loci previously identified in ASD and schizophrenia has been demonstrated (Thapar et al., 2016; Gudmundsson et al., 2019). Overall, whereas the high heritability of ADHD can be estimated with confidence, the underlying genetic factors are complex, both common and rare variants play an important role in the susceptibility to the disorder.

Although genetic effects are more pronounced in the etiology of ADHD, 22% of phenotypic variance can be attributed to environmental factors (Faraone et al., 2005; Nikolas and Burt, 2010). In the case of certain genes, the gene expression and thus the phenotypic trait are influenced by environmental and consequential epigenetic effects. Epidemiological studies have identified several environmental risk factors in the background of ADHD, which include maternal substance use, stress, the presence of environmental toxins in the prenatal or perinatal period, and low birth weight. Similarly, ADHD risk was increased in preterm birth, in the presence of psychosocial adversities during early childhood, or in the case of suboptimal nutritional factors (Loche and Ozanne, 2016). These results significantly overlap with the results of epigenetic studies, although the risk factors associated with the disorder are not necessarily causal. Studies to date have shown that maternal smoking, alcohol and substance use, and also suboptimal nutrition can have a significant effect on transcription. In adolescence, increased stress, trauma, and abuse play a role in the epigenetic modifications. Besides the epigenetic effects, which are exerted through chemical modifications of DNA molecules, such as cytosine methylation, histone modifications, or RNA-mediated modifications, there are candidate genes for which the presence of a certain variation in itself is associated with increased environmental vulnerability to ADHD. Examples include interactions between dopamine receptor D4 (*DRD4*) and maternal smoking (Pluess et al., 2009), dopamine transporter (*DAT1*) and maternal alcohol consumption during pregnancy (Brookes et al., 2006), or between serotonin transporter (*5HTT*) and adverse psychosocial events (Müller et al., 2008) (see review Palladino et al., 2019).

### The Genetic Background of the Core Symptoms and the Lifespan Perspective, Differences Between Attention-Deficit/Hyperactivity Disorder Traits and Diagnosis

The clinical diagnosis of ADHD is based on the lifelong presence of the core symptom domains such as inattention, hyperactivity, and impulsivity, which are associated with functional impairment in daily life, and the onset of these symptoms in childhood. Although the heritability of ADHD dimensional traits is somewhat lower than that of ADHD itself, clinically diagnosed ADHD and population traits of the disorder overlap significantly in terms of genetic risk (Demontis et al., 2019).

Research by Taylor et al. (2019) used a joint categorical/continuous twin method to estimate the genetic correlation between psychiatric diagnoses (ADHD, Tic disorders, obsessive–compulsive disorder, anxiety, major depressive disorder, and schizophrenia), and the corresponding continuous traits of these disorders offered a better understanding of the partial discrepancy. Subsequent to examining the association between PRS for each disorder and associated traits, additional analyses were done after excluding individuals diagnosed with the relevant psychiatric disorder. Authors found that ADHD PRS was associated with ADHD population traits [ß (SE) = 0.27 (0.03)] at the age of 9 years. The phenotypic correlation between ADHD diagnosis and ADHD-related traits was 0.52 (0.50–0.54), and both ADHD dimensions displayed moderate phenotypic (mean estimate: 0.49; range: 0.45–0.53) and genetic (mean estimate: 0.53; range: 0.49–0.57) correlations with ADHD diagnosis. Cross-trait analyses yielded a correlation of 0.47 (0.43–0.51) in monozygotic (MZ) and 0.17 (0.13–0.21) for dizygotic (DZ) samples (Taylor et al., 2019).

Besides the high genetic correlation observed between ADHD case–control status and ADHD-related traits (Bidwell et al., 2017; Demontis et al., 2019), the symptom dimensions of ADHD also show a significant genetic correlation with each other [rg (SE) = 0.73 (0.08)] (Ebejer et al., 2013; Bidwell et al., 2017). Consequently, distinguishing between the clinical presentations of ADHD is of practical importance due to the differences in the lifelong continuation of the symptoms, the nature of the related functional impairment, and also the vulnerability to other psychiatric comorbidities which impact treatment decisions (Lahey et al., 2002). Nevertheless, current genetic results are more in favor of a trait-based, rather than a cutoff (the presence or absence of ADHD diagnosis) approach, whereby the symptoms are considered as quantitaive traits along a dimensional spectrum, with ADHD itself corresponding to extremes of the spectrum in which the symptom is present with sufficient intensity, and continuity over time required for the diagnosis (Larsson et al., 2012). Based on current studies, ADHD PRS values, that is, an individual estimate of overall SNP effects, have been consistently associated with ADHD diagnosis and functions in a dose-dependent manner (Ronald et al., 2021). ADHD PRS was significantly associated with ADHD dimensional symptom severity scores both in clinical and population samples (Albaugh et al., 2019; Burton et al., 2019; Stojanovski et al., 2019; Nigg et al., 2020) in parent-report (Nigg et al., 2018), self-report (Burton et al., 2019), and also teacher-rated scales (de Zeeuw et al., 2020) (see review Ronald et al., 2021). As gene–environment interactions play an important role in the background of ADHD symptom severity, the study of Selzam et al. (2019) using DZ twin samples and comparing between-family and within-family factors is particularly interesting. The between-family ADHD PRS effect, which was estimated independent of the within-family effect, significantly predicted more ADHD traits. The within-family ADHD PRS effect showed that, within pairs, the twin with higher ADHD PRS had more ADHD traits than their cotwins. Another remarkable finding is that the ADHD genome-wide polygenic score within-family prediction was significantly lower than between-family prediction for educational achievement, and the between-family ADHD PRS on educational achievement was significantly reduced when socioeconomic status was controlled for, but remained significant (Selzam et al., 2019).

Based on the results of recent studies, ADHD PRS is also positively associated with ADHD-related traits. In two studies, significant association was found with both symptom dimensions (inattention and hyperactivity/impulsivity) (Burton et al., 2019; Taylor et al., 2019), whereas in two other studies, association only with hyperactivity/impulsivity was reported (Sudre et al., 2020; Vuijk et al., 2020). The heterogeneity of the symptoms, the dynamic changes in the ADHD symptom presentation over the lifetime of the patient, or the presence of subthreshold symptoms in the other symptom domain, which may not cause functional impairment at a given life period, are also consistent with this result (5th ed.; DSM-5; American Psychiatric Association, 2013; Stojanovski et al., 2019).

Several genetic findings, such as the genetic correlation for both common and rare variants, significantly overlap with ASD (Grove et al., 2019; Satterstrom et al., 2019), or the correlation between ADHD and lower IQ underscored the neurodevelopmental nature of ADHD (Frazier et al., 2004; Pinares-Garcia et al., 2018). A meta-analysis of longitudinal studies found that at least 15% of children diagnosed with ADHD continue to meet diagnostic criteria at age of 25, and further 50% of the cases reach only partial remission still causing impairment in their everyday life (Faraone et al., 2006). As the heritability of ADHD is stable across the lifespan (Kuntsi et al., 2005; Kan et al., 2013; Brikell et al., 2015), there is an emerging research interest in capturing persistence at the genetic level. In a well-powered meta-analysis of GWAS results, which included 17,149 cases and 32,411 controls, evolving childhood ADHD and adult ADHD cases were analyzed separately and jointly (Rovira et al., 2020). Nine independent loci were identified that overlapped in the childhood and adult ADHD groups, all of which were common variants playing a role in certain stages of brain development such as neuronal migration, myelination, or diencephalon development. These hits did not overlap with the results of previous candidate gene studies. In this study, the genetic correlation between childhood ADHD and adult persistent ADHD was found to be remarkably high (rg = 0.81, CI: 95% 0.64–0.94). Further analysis was aimed at investigating whether latent persistent cases genetically differed from the non-persistent cases; however, variants specifically associated with ADHD persistence could not be identified (Rovira et al., 2020), and persistence was also independent from gender (Caye et al., 2016). Given the familial aggregation of ADHD persistence demonstrated in earlier studies, the notion arises that rare variants and also gene–environment interactions may also play a role in the genetic background of persistence. Since persistence is a dynamic process over time, longitudinal studies with the potential of examining remitting cases could provide more insight into the potential gene by age interactions (Kuntsi et al., 2005; Chang et al., 2013; Thissen et al., 2015).

The evolutionary perspective of the ADHD symptoms raises the question why these symptoms have not bred out through the processes of natural selection despite that they are related to adverse functional outcomes (Keller and Miller, 2006; Nesse, 2006). One explanation for this – also known as the mismatch theory (Crawford and Salmon, 2002; Durisko et al., 2016) or the anachronism of ADHD (Arcos-Burgos and Acosta, 2007) – could be that changes in human societies have occurred so rapidly that they have outpaced the much slower evolutionary changes required to select for these traits (Jensen et al., 1997). Another potential approach is the natural positive selection theory (Thagaard et al., 2016), which suggests that ADHD traits may have been beneficial in certain circumstances. For example, in a hunter-gatherer environment which is typically characterized by the depletion of resources, and also time-critical and novel-rapidly changing conditions, hyperactivity could have been advantageous in spotting new opportunities or migrating toward better climates, whereas impulsivity as related to the response-readiness and the ability to fight-or-flee, and inattention, as a high-scanning behavior could have likely been adaptive under these environmental conditions (Jensen et al., 1997). Results of two studies (Ding et al., 2002; Wang et al., 2004) support this theory as they found that the increased 7R frequency in *DRD4* gene related to ADHD may be the result of positive selection since it is associated with both ADHD and the personality trait of novelty seeking (Benjamin et al., 1996; Gizer et al., 2009). An alternative hypothesis for this positive selection is simply that ADHD traits have persisted because they increased reproductive success, as it is well known that compared with individuals without ADHD, patients are more likely to be younger at first sexual intercourse, to have more sexual partners and to be involved in teenage parenthood (Barkley et al., 2006; Flory et al., 2006; Østergaard et al., 2017; Esteller-Cucala et al., 2020).

The findings in ASD substantiating that the PRS for ASD is positively correlated with general cognitive ability in the general population (Clarke et al., 2016) are also thought-provoking in an evolutionary context, especially given the considerable overlap between the ASD and the ADHD phenotype. However, in their study, Clarke et al. (2016) did not find consistent evidence in support of the association between the polygenic risk for ADHD and cognitive function. Beyond the theoretical hypotheses, behavioral observations are of major importance. Arildskov et al. (2021) applied behavioral tests to mimic ancestral environmental conditions. Studying school age children in a response-readiness laboratory test, continuous ADHD traits (measured by the ADHD-RS-IV) were not found to be related to the test performance. Nevertheless, it is important to emphasize the limitation of such studies, namely that it is difficult to create a situation mimicking all ancestral environmental conditions that can have all potential effects on the performance; moreover, there could be other ADHD-related advantages of these traits, which may not be in the focus of the given study.

### Attention-Deficit–Hyperactivity Disorder and Psychiatric Comorbidities

It is estimated that around 60–100% of children with ADHD also exhibit one or more comorbid disorders that often continue into adulthood complicating the diagnosis and the treatment (Biederman et al., 1993; Gillberg et al., 2004). When compared with non-ADHD subjects, significantly higher rates of comorbid major depression, dysthymia, bipolar disorder, anxiety disorders, substance use disorders, and personality disorders were consistently reported in adults with ADHD (Jacob et al., 2007; Sobanski et al., 2007; Cumyn et al., 2009; Asherson et al., 2014; Perroud et al., 2014; Bitter et al., 2019). Results of cross-disorder studies show that there is a significant genetic overlap between the genetic backgrounds of mental disorders, the so-called general psychopathological factor accounting for 10–57% of the phenotypic variance (Lahey et al., 2011; Caspi et al., 2014; Pettersson et al., 2016; Waldman et al., 2016; Selzam et al., 2018; Allegrini et al., 2020; Brikell et al., 2020). One possible explanation is that the different clinical phenotypes are the consequence of the highly pleiotropic effects of the genetic variants that contribute to the risk for developing psychiatric disorders. Another hypothesis is that these variants primarily define traits that are present in a subclinical form in many diseases, whereas in the case of other disorders, their expression becomes more dominant (Plomin et al., 2009). For example, in terms of psychiatric vulnerability, ADHD PRS has been shown to be positively associated with sensation-seeking, risk-taking behavior, and irritability (Du Rietz et al., 2018; Riglin et al., 2019; Nigg et al., 2020). PRS differences within the ADHD groups based on the clinical presence of emotional lability or different impulsivity factors suggest additional clinical subtypes with different genetic risks (Grimm et al., 2020; Nigg et al., 2020).

Observations of family and twin studies indicated a significant genetic overlap between ADHD and ASD (Rommelse et al., 2010; Ghirardi et al., 2018), schizophrenia (Larsson et al., 2013), bipolar disorder (Faraone et al., 2012), major depression (Faraone and Biederman, 1997), and antisocial personality disorder (Christiansen et al., 2008). The ADHD PRS results from GWAS are only partially consistent with these findings. ADHD PRS showed a significant positive association with the general psychopathology factor in children (Riglin et al., 2019), the ADHD + bipolar disorder group was characterized by a higher PRS (only compared to the control group), and no association was found with schizophrenia. In depression, anxiety, neuroticism, and also eating disorders, the results were contradictory, in many cases showing age-related effects (Ronald et al., 2021). In a GWAS mega-analysis of the PGC Cross-Disorder Group (Lee et al., 2019), data from 232,964 cases and 494,162 controls were analyzed across eight disorders: ADHD, anorexia nervosa, ASD, schizophrenia, bipolar disorder, major depression, obsessive–compulsive disorder, and Tourette syndrome. They identified 109 pleiotropic loci confirming the significantly shared genetic background of psychiatric disorders. ADHD showed the strongest SNP-based genetic correlation with major depression (rg = 0.44), followed by a positive association with neurodevelopmental disorders (ASD rg = 0.37, Tourette syndrome rg = 0.27) ascertained by LD score regression analysis.

It is important to note that in the five studies which investigated either diagnosed autism or autistic traits in most cases, ADHD PRS did not predict these traits (Jansen et al., 2020; Serdarevic et al., 2020; Torske et al., 2020), only one study (LaBianca et al., 2021) (on autism) reported a significant positive association with the ADHD PRS, and another study (Serdarevic et al., 2020) reported a significant positive association only in male participants. ADHD PRS did not associate with other neurodevelopmental conditions; however, a forward-looking result is that in non-ADHD disorders, ADHD PRS appears to have transdiagnostic utility in characterizing subgroups of individuals with early-onset symptoms. For example, although ADHD PRS did not associate with schizophrenia, within a schizophrenia sample, it is associated with the cognitive trajectory from adolescence into adulthood, showing the strongest association with the subgroup presenting with the earliest preadolescent cognitive impairment (Dickinson et al., 2020).

The correlation between ADHD and internalizing, externalizing, and neurodevelopmental diseases has been targeted in several studies (Du Rietz et al., 2018; Demontis et al., 2019; Lee et al., 2019). In a large longitudinal study (Du Rietz et al., 2021b), ADHD indicated a significant association with all three groups (*r* = 0.67–0.75); however, after correcting for the general psychopathology factor, only the association with neurodevelopmental disorders remained moderately strong (*r* = 0.43, 95% CI: 0.42–0.45), which is largely influenced by genetic factors. The association with externalizing disorders was lower (*r* = 0.25, 95% CI: 0.24–0.27), which is largely influenced by environmental effects, and there was no significant association with internalizing disorders. Overall, current studies suggest that although the genetic overlap between ADHD and other psychiatric disorders is significant, it could likely be explained by general psychopathology factors, and the role of unique genetic effects may be plausible mostly between ADHD and other neurodevelopmental disorders.

### Biomarkers and Potential Endophenotypes

The diagnosis of ADHD is hindered by the heterogeneity of the disease, variability of symptom presentation over time, and subjectivity of symptom severity, as potential confounding factors; therefore, interest in predictive biomarkers that could aid the diagnosis, prognosis, and assessment of the response to pharmacological interventions has increased substantially (Faraone et al., 2014; Mehta et al., 2020). Endophenotypes (Gottesman and Gould, 2003), a subtype of biomarkers (Biomarkers Definitions Working Group, 2001), are quantitative indicators of the biological processes underlying the disease rather than of the clinical phenotypes. These measures should be quantifiable, state independent, they are expressed regardless of whether the disorder is manifest, and they are more prevalent in the unaffected relatives of patients than in the general population. Genetic determination is a prerequisite for the definition of endophenotypes, that is, the endophenotype is heritable, the endophenotype and the disorder are associated within the family, it must show association and/or linkage with one or more of the candidate genes, and the endophenotype should mediate the association and/or the linkage between the candidate gene and the disorder. The hope of identifying endophenotypes has reoriented research interest to candidate gene studies.

Of the ADHD candidate genes linked to dopaminergic, noradrenergic, and serotonergic neurotransmitter systems (Gizer et al., 2009), most results are available for *DAT1* and *DRD4* in terms of various endophenotypes (Faraone et al., 2014). Summary of findings is provided in Table 1.

**TABLE 1:** Association of VNTR polymorphisms in candidate genes DRD4 and DAT1 with various ADHD-related neuropsychological, eletrophysiological and neuroimaging measures emerging as putative endophenotypes.

Imaging modality	Putative endophenotypes		References
	**Association with DRD4 exon 3 VNTR**	**Association with DAT1 3’UTR VNTR**	
Neuropsychological test	Cognitive endophenotype studies found an association between DRD4 7R allele and processing speed, cognitive impulsiveness and attention shift, while the absence of the 7R allele was linked to high reaction time variability. No association found with the behavioral indicators of response inhibition.	Contradicting results. The most evidence has been found in relation to response inhibition, verbal and visuospatial working memory, executive functions and sustained attention. 10R allele showed a positive association with higher commission error and reaction time variability on CPT and SART tests.	Langley et al., 2004, see reviews: Barnes et al., 2011; Kebir and Joober, 2011; Faraone et al., 2014; Mehta et al., 2020.
Electrophysiology: event related potential (ERP) parameters	Children carrying the risk allele demonstrated lower Cue-P300 and contingent negative variation (CNV) event-related potential amplitudes suggesting a possible specific effect on attentional orienting and response preparation processes.	Reduced NoGo anteriorization (NGA) in Go/No-Go task (indicating impaired cognitive response control) in adult ADHD 9R carriers. Decreased error positivity (Pe) amplitude and feedback anticipatory negativity (SPN) in feedback-based learning task in children 10R/10R -carriers vs. 9R carriers.	Althaus et al., 2010; Dresler et al., 2010; Albrecht et al., 2014.
Electrophysiology: quantitative EEG measures	Inreased frontal theta power and decreased global beta power in children 7R carriers. Adult 7R carriers (parents): similar beta2 power in ‘eyes closed’ condition, but decreased beta2 power in ‘eyes open’ and CPT conditions.	Medication-related EEG changes (single dose of 10mg methylphenidate) of increased central and parietal beta power, and decreased right frontal theta power and lower theta/beta ratios in children 10R/10R carriers vs. 9R carriers (in sustained attention task).	Loo et al., 2003, 2010.
Structural brain imaging	Decreased cortical volume in DLPFC. (Decreased superior frontal and cerebellar cortex volumes associated with the 7R allele in ADHD were described in adult patients.) An emerging hypothesis from a longitudinal study is that carrying the 7R allele may be associated with cortical development.	Smaller nucleus caudatus volume in 10R/10R homozygous children compared to 9R/10R heterozygotes. Enlarged striatal volume in adults carrying 9-6 haplotype (risk haplotype for adults).	Shaw et al., 2007; Monuteaux et al., 2008; Shook et al., 2011; Onnink et al., 2016, review: Klein et al., 2017.
Functional braing imaging	DRD4 VNTR may play a role in the development of white matter connectivity as well.	Decreased dorsal striatum (nucleus caudatus) activity described in adolescent ADHD patients in a reward processing paradigm. Increased activity in frontal, medial, and parietal regions, in left striatum, and right dorsal premotor cortex compared to 9R carriers in Go/No-Go task (in children and adolescent samples).	Paloyelis et al., 2012; Takeuchi et al., 2015; see review: Klein et al., 2017.

The dopamine transporter gene (*DAT1*) codes for the solute carrier protein responsible for the reuptake of dopamine from the synaptic cleft. Gene expression is most pronounced in the striatum. The most intensively studied variant of *DAT1* is variable tandem repeats (VNTRs) of 40 base pairs located at the 3’untranslated region (3′UTR) of which 10-repeat (10R) and 9-repeat (9R) alleles occur most frequently. An additional VNTR polymorphism located in intron 8 containing 5R and 6R alleles, which has also been associated with increased susceptibility to ADHD, is also being studied as a haplotype. Interestingly, based on the previous studies, whereas the 10R/10R genotype and 10/6 haplotypes are likely to be risk factors in children (Brookes et al., 2006; Asherson et al., 2007), the 9R/9R genotypes and 9/6 haplotypes were associated with the disease in adult patients with ADHD (Franke et al., 2010). The *DRD4* gene is predominantly expressed in the anterior cingulate cortex (and orbitofrontal cortex), a brain region of major importance for attentional and inhibitory processes. The most commonly studied polymorphism of the *DRD4* gene is the 48-bp VNTR in exon3, the 7R allele of which is linked to an increased risk of ADHD in the Caucasian population (Wu et al., 2012).

The heterogeneity of the ADHD symptoms and the complexity of its genetic architecture have put endophenotype studies in a new context in recent years, drawing the attention to the difficulty of defining the genotype and the phenotype, let alone creating a bridge between them, which would in fact be key to a more complete understanding of the genetic or biological determinants and the clinical phenotype. The challenges associated with and the implications of linking genes to structural and functional variations in the brain systems responsible for cognition and emotion are considerable.

A major difficulty is that the methodological aspects of neuroimaging (Shaw et al., 2007; Monuteaux et al., 2008; Shook et al., 2011; Paloyelis et al., 2012; Takeuchi et al., 2015; Onnink et al., 2016; Klein et al., 2017), electrophysiological (Loo et al., 2003, 2010; Althaus et al., 2010; Dresler et al., 2010; Albrecht et al., 2014), and neuropsychological (Langley et al., 2004; Barnes et al., 2011; Kebir and Joober, 2011; Faraone et al., 2014; Mehta et al., 2020) studies, which served as potential endophenotypes, differ from the methodological preferences of genetic studies. For example, as the case–control design by gender, age, and education is of high importance for cognitive performance, the majority of these studies have low sample sizes; moreover, the heterogeneity of methodological parameters of the paradigms applied in the studies resulting in task-dependent changes in cognitive performance constitutes a limitation to the aggregation of certain phenotypic variables.

Chauvin et al. (2021) offer an overarching approach to this issue facilitating the comparison between task paradigms that are shared across multiple cognitive functions, resembling a cognitive core, from those that are task-specific (Konrad and Eickhoff, 2010; Lin et al., 2014). The results of the study (Chauvin et al., 2021) are promising for the endophenotype approach, as ADHD siblings displayed a task connectivity modulation profile that is an intermediate between diagnosed ADHD siblings and control participants; namely, they showed a similar degree of task generic connectivity modulation as controls, but significantly more task-specific connectivity modulation than ADHD probands. In the context of neuroimaging techniques, it is also important to mention that even for the same genetic mechanism, the effect size can vary widely depending on the imaging target measure (structural variation, functional activation, or functional connections) examined (Hariri et al., 2002; Meyer-Lindenberg and Weinberger, 2006).

The difficulty in selecting the optimal psychological test and test indicators is another factor that adds to the complexity of endophenotype studies. Neuropsychological tests that are characterized with higher effect sizes in patients with ADHD (Bálint et al., 2009) – such as the continuous performance tests (Conners et al., 2003) or the standardized neuropsychological measures, for example, Stroop task (Golden, 1975), the Digit Span subtest of WAIS (Wechsler, 1981) or the Wisconsin Card Sorting Test (Heaton et al., 1993) – are also widely used in endophenotype investigations; however, the results are inconsistent (Kebir and Joober, 2011; Faraone et al., 2014). A study by Acosta-López et al. (2021) offers a promising approach to overcome the gap between the genotype and the phenotype, whereby applying a family-based design and using an extensive test battery of neuropsychological tasks (Stroop test, Cross-Out-Squares Test, and Trail Making Test) and also reaction time-based task paradigms (Conners’ Continuous Performance Test and Go/No-Go Tasks (Jiménez-Figueroa et al., 2017; Jimenez-Figueroa et al., 2020); temporal processing is evaluated as a potential endophenotype in ADHD. Estimating the effect sizes for neuropsychologically based variables related to a cognitive mechanism (e.g., temporal processing) in a case–control design and the parallel estimation of the heritability of these variables within one sample group can provide variables which are likely to be relevant to both clinical (neuropsychological) and genetic perspectives.

The current results of electrophysiological studies in terms of endophenotypes are still limited. Based on the recently available twin and family studies, the resting state EEG measures are related to higher heritability rates compared with event-related potentials (Iacono, 2014). Iacono’s publication (Iacono et al., 2017) reviews a variety of electrophysiological measures and classifies them as the biomarkers or putative endophenotypes. For ADHD, moderate evidence was considered for error-related negativity (ERN) amplitude (Albrecht et al., 2008; Anokhin et al., 2008; McLoughlin et al., 2009), and suggestive evidence was considered for very low-frequency EEG activity (Tye et al., 2012), increased theta and delta power (Loo et al., 2010), and decreased beta power (Loo et al., 2010; Rudo-Hutt, 2015). From event-related EEG measures, ITPC (intertrial phase coherence) (McLoughlin et al., 2014), No-Go N2 amplitude observed in the flanker task (Albrecht et al., 2008; McLoughlin et al., 2009), and the extensively investigated amplitude of the P300 component (van Beijsterveldt and van Baal, 2002; Malone et al., 2014) have suggestive evidence in terms of the endophenotype criteria (Iacono et al., 2017). Interestingly, the ratio of theta to beta resting EEG power may constitute only a biomarker for ADHD, as it does not appear to be genetically influenced (Snyder and Hall, 2006; Arns et al., 2013).

Notably, there are additional emerging approaches such as the investigation of oscillation potential change related to a trait, since the hierarchy of brain oscillations has remained remarkably preserved during the course of mammalian evolution (Buzsáki et al., 2013). Examples include the examination of oscillatory activity in psychiatric disorders frequently associated with impaired language skills such as schizophrenia (Murphy and Benítez-Burraco, 2016) or ASD (Benítez-Burraco and Murphy, 2016). Considering that the symptoms of inattention and hyperactivity often coexist with language problems in both clinical and community samples (Hawkins et al., 2016) and the reciprocal presence of the disorder-related traits between ASD and ADHD symptoms (Baixauli-Fortea et al., 2019), examining oscillopathic alterations could be a promising opportunity to construct successful endophenotypes also in patients with ADHD. It is highly important in terms of potential electrophysiological endophenotypes to emphasize that during development, the longitudinal stability of the different EEG measures is different; consequently, the relationship between the gene and the endophenotype may not be constant across the development; therefore, the utility of the endophenotype may be limited to certain developmental periods (Poil et al., 2014; Giertuga et al., 2017).

There are also several methodological issues associated with genotyping. For example, the notion that endophenotypes are genetically less complex than psychiatric disorders is controversial and not necessarily true (Roffman, 2019). Additionally, the potential gene–gene interactions or the effect of environmental factors on gene transcription are often neglected aspects. As genetic studies have moved from the univariate candidate gene risk polymorphism approach to multimarker analysis, it is reasonable to examine the relationship between multiple genetic variables and ADHD phenotypes. To explore the genetic background of endophenotypes in genetic factors associated with psychiatric phenotypes (Iacono et al., 2017; Dick, 2018), the association between ADHD PRS and neuroimaging or neuropsychological indicators has been increasingly investigated in recent years. Of the neuropsychological indicators, the association between working memory and ADHD PRS is the most consistent result (Nigg et al., 2018; Hermosillo et al., 2020; Shen et al., 2020; Sudre et al., 2020; Torske et al., 2020; Vuijk et al., 2020); nevertheless, there are also positive findings for focused attention, delay discounting, and vigilance or arousal, whereas surprisingly, no association was found with indicators of executive functions (such as response inhibition). Due to the significant clinical overlap between ADHD and other psychiatric disorders, there is an increasing effort to define clinical phenotypes that are etiologically related. For example, considerable attention is paid to the investigation of developmental endophenotype with the intention of also elucidating whether these indicators reflect disease- or condition-specific biological pathways or may be more linked to general psychopathological processes (Gui et al., 2020).

In summary, although there have been several studies aimed at identifying endophenotypes, they still represent early attempts yielding contradictory results, and so far, none of the indicators have met all criteria required in the definition. Studies to date have had small sample sizes, differed in study design, and have been inconsistent in the interpretation of the association with the genotype. The lack of evidence substantiating a causal relationship between the genetic risk factors and the phenotype should also be highlighted, given that only few studies conducted a mediation analysis are available. A further limitation of endophenotype studies is the difficulty of capturing gene–environment interactions which are of great importance in ADHD and emphasizes the need for further longitudinal studies.

### Therapeutic Aspects

Several biological mechanisms, including dopaminergic, serotoninergic, and glutamatergic signaling, have been implicated in the etiology of ADHD (Bonvicini et al., 2016). Medications approved by the US Food and Drug Administration (FDA) (Multiple authors, 2018) for the treatment of ADHD are stimulants such as methylphenidate (MPH) and amphetamines, and non-stimulant medications such as the selective noradrenaline reuptake inhibitor atomoxetine (ATX), the alpha-2 agonists guanfacine and clonidine, and the recently approved serotonin noradrenaline-modulating viloxazine. Pharmacogenetic studies are primarily focused on predicting drug responsiveness, and given the heterogeneity of drug responses, they are also aimed at exploring whether genetic determinants determine individual treatment response (Elsayed et al., 2020). This is even more relevant considering that the current pharmacological ADHD treatment is effective only in about 70% of the cases (Jensen et al., 2007), and the effect size is 30–50% decreased in adults compared with children (Cortese et al., 2018a). Significantly, fewer studies target the investigation of the effective dose and the optimal drug dose for undesired side effects, despite them being highly relevant for therapeutic adherence (Storebø et al., 2018; Brown et al., 2019; Bousman et al., 2021).

Although only a small proportion of the vulnerability to ADHD is linked to a single gene, given the monoaminergic attack points of drugs, pharmacogenetic studies initially focused on the potential associations of candidate ADHD genes and drug efficacy. Most data are available for methylphenidate and atomoxetine. Results from a meta-analysis of childhood and adolescent ADHD indicated significant associations between several common variants, namely *DAT1*, *DRD4* VNTRs, and SNPs in the *ADRA2A*, catechol-O-methyltransferase (*COMT*), and noradrenaline transporter (*SLC6A2*) genes, and MPH responsiveness (Myer et al., 2018). In addition, the latrophilin-3 gene (*ADGRL3* = *LPHN3*) carrying the G allele has also been correlated with MPH treatment response (Arcos-Burgos et al., 2010; Labbe et al., 2012; Özaslan et al., 2021). In adult patients with ADHD, Contini et al. (2012, 2013) found no associations with *DRD4*, *COMT*, serotonin receptor 1B (*HTR1B*), tryptophan hydroxylase (*TPH2*), dopamine β-hydroxylase (*DBH*), *5HTT*, and synaptosomal-associated protein 25 (*SNAP25*) genes, only with *DAT1* (Contini et al., 2012; see review Contini et al., 2013). In contrast, in a pharmacogenetic review and meta-analysis specifically targeting *DAT1*, no association was detected between the 40-bp VNTR and the MPH response (Bonvicini et al., 2016). An additional interesting finding is the possible correlation between *SNARE* complex polymorphism and MPH responsiveness in an adult sample (da Silva et al., 2018).

In the case of atomoxetine, significant correlation was found between the response status in childhood ADHD and *DBH* and *SLC6A2* in a Chinese sample, and later in a broader sample (Yang et al., 2013; Fang et al., 2015; Gul et al., 2021). Further pharmacogenetic studies of atomoxetine target the cytochrome P450 polymorphisms to determine the possible genetic factors for poor, extensive, high, and ultrarapid metabolism (Michelson et al., 2007; Trzepacz et al., 2008; Fijal et al., 2015, see review Yu et al., 2016).

A summary of pharmacogenetic studies (Mick et al., 2006; Kooij et al., 2008; Nemoda et al., 2009; Ramoz et al., 2009; Contini et al., 2010, 2011, 2012; Johnson et al., 2013; Yang et al., 2013; Fang et al., 2015; Hegvik et al., 2016; Gomez-Sanchez et al., 2017; Pagerols et al., 2017; Angyal et al., 2018; Huang et al., 2018; Naumova et al., 2019; Bonvicini et al., 2020; Gul et al., 2021; Yuan et al., 2021) assessing the most investigated candidate genes is provided in Table 2.

**TABLE 2:** Summary of the pharmacogenetic studies assessing the most extensively investigated candidate genes and/or using genome-wide method in ADHD patients on methylphenidate and atomoxetine treatment.

Medication	Results of candidate genes studies			Results of GWAS studies
	Gene	Polymorhism(s)	Association between the genotype and drug response	
Methylphenidate	DAT1	3’ UTR 40bp VNTR	Conflicting results. Reduced efficacy for 10R homozygotes: *Myer et al., 2018*. No association: Gomez-Sanchez et al., 2017. Single 10R variation associated with increased MPH response compared to 10/10 homozygosity in adults: Kooij et al., 2008. No association in adults: Mick et al., 2006; Contini et al., 2010; *Bonvicini et al., 2016*; *Hegvik et al., 2016*.	No genome-wide significant hits identified on methylphenidate response: Pagerols et al., 2018; Mick et al., 2008 (limited sample size, N<200).
		Intron 8 VNTR	Lack of 6R homozygosity associated with faster MPH response over time: Gomez-Sanchez et al., 2017. No association in adults: Contini et al., 2010.	
	DRD4	Exon 3 48bp VNTR	Homozygous 4 repeat genotype associated with improved MPH response. No association between 7R genotype and MPH response: *Myer et al., 2018*. Improved MPH response with 7R allele: Naumova et al., 2019. No association with genotype: Gomez-Sanchez et al., 2017. No association in adults: *Hegvik et al., 2016*. Modulation of MPH response across the lifespan, differential associations depending on age and population: *Bonvicini et al., 2020*.	ADHD PRS associated with higher symptom improvement following stimulant treatment: Zhong et al., 2020.
		120bp promoter duplication	Decreased response with homozygous short allele promoter duplication: Gomez-Sanchez et al., 2017.	No correlation between 23 genes identified as targets of methylphenidate and ADHD GWAS gene-level summary statistics. Within the loci associated with ADHD 5 druggable genes encode proteins interacting with FDA-approved or clinical trial drugs:PTPRF, TIE1, MPL, SLC6A9 and KCNH3: Hegvik et al., 2019.
	ADRA2A	rs1800544	G allele associated with improved response compared with patients carrying C allele: *Myer et al., 2018*. GG genotype associated with improved MPH response: Huang et al., 2018. G allele associated with improvement in inattention symptoms: *Yuan et al., 2021*. No association with response: Gomez-Sanchez et al., 2017. G allele associated with MPH non-response in adults: Hegvik et al., 2016. No association in adults: Contini et al., 2011; *Hegvik et al., 2016*.	
	COMT	rs4680	Val/Val genotype associated with improved response compared with Met allele carriers: *Myer et al., 2018*. No association: Pagerols et al., 2017, adults: Contini et al., 2012; *Hegvik et al., 2016*.	
	CES1	8 SNPs	No association between genotype and responder status: Johnson et al., 2013; Nemoda et al., 2009. No association in adults: *Hegvik et al., 2016*.	
	LPHN3	rs5661665, rs1947274	No significant association: *Myer et al., 2018*.	ADHD and ASD PRS not associated with stimulant initiation, discontinuation or switch. No GWAS hits were found for stimulant initiation or discontinuation: Brikell et al., 2021.
		rs6551665	G allele carriers exhibited better response in the inattentive symptom domain: Arcos-Burgos et al., 2010. G allele associated with poor response: Labbe et al., 2012. GG genotype associated with poor drug (MPH and ATX) response: Özaslan et al., 2021. No association in adults: *Hegvik et al., 2016*.	
	SLC6A2	rs5569	G/G genotype associated with improved response compared to A allele carriers: *Myer et al., 2018*. No significant association: Gomez-Sanchez et al., 2017.	
		rs28386840	T allele associated with improved response: Angyal et al., 2018; *Myer et al., 2018*; *Yuan et al., 2021*. No significant association: Gomez-Sanchez et al., 2017. No association in adults: *Hegvik et al., 2016*.	
Atomoxetine	DBH	rs2519154	Decreased response with C allele: Fang et al., 2015.	No association with drug (MPH+ATX) response neither in SNP nor in gene-level analyses in a study investigating neurodevelopmental genes identified in ADHD. ADHD PRS not associated with symptom improvement following ATX treatment (possibly due to the limited sample size): Zhong et al., 2020.
	SLC6A2	rs3785143	Non-response linked to the presence of T allele: Yang et al., 2013. Better treatment response and more side effects in children with both rs3785143 and rs12708954 heterozygous genotype than in patients with wide type: Gul et al., 2021.	
		108 SNPs	No association for any SNP: Ramoz et al., 2009.	
	CYP2D6	allelic variants related to poor, intermediate, normal and ultrarapid metabolization	Poor metabolizers are more likely to experience improvement in ADHD symptoms compared to extensive metabolizers: Michelson et al., 2007; Trzepacz et al., 2008. No association: Ramoz et al., 2009. Poor metabolizers are at increased risk of having side effects compared to non poor metabolizers: Michelson et al., 2007; Trzepacz et al., 2008; Fijal et al., 2015.	
	ADRA1A	3 SNPs	No effects on drug response: Yang et al., 2013.	
	ADRA2A	rs1800544	GG haplotype linked to non-remission status (not significant after correction for multiple comparisons): Yang et al., 2013.	

*DAT1, dopamine transporter; DRD4, dopamine receptor D4; ADRA2A, Adrenergic α2A Receptor; COMT, catechol-O-methyltranspherase; CES1, Carboxylesterase; LPHN3, latrophilin-3 gene; NET, Norepinephrine Transporter; DBH, dopamine β-hydroxylase; CYP2D6, Cytochrome P-450 2D6; ADRA1A, Adrenergic α1A; MPH, methylphenidate; ATX, atomoxetine. Meta-analyses and/or reviews are highlighted in italics.*

In terms of pharmacogenetic studies, genome-wide association studies, which are not hypothesis-driven, do not overlap with the results of candidate gene studies. In a GWAS study with the highest sample size and statistical power available to date (Hegvik et al., 2019), no association was found between ADHD and the effectiveness of FDA-approved first-line pharmacological agents, suggesting that they may exert their effect through mechanisms different from the ones underlying ADHD. One of the main advantages of GWAS is that they better capture the heterogeneity of ADHD and the cross-disorder nature of the characteristic traits, thereby facilitating the identification of potential new therapeutic options. It opens the possibility of drug repurposing, that is, using other drugs that have been shown to be effective in other psychiatric disorders. The successful utilization of the metabotropic glutamate receptor activating fasoracetam, an agent previously used in vascular dementia, in ADHD adolescents in whom the glutamatergic signaling is affected serves as a good example of drug-repurposing (Elia et al., 2018). The high psychiatric comorbidity of ADHD, especially in adulthood, and also the significant symptomatic and genetic overlap with other psychiatric disorders raise the possibility of another therapeutic approach whereby the medication is selected based on the presence of a characteristic trait. Symptoms that are often associated with ADHD such as emotional or affect lability for which the efficacy of current agents is inadequate can serve as the examples for this approach. Summary of findings using genome-wide method (Mick et al., 2008; Pagerols et al., 2018; Hegvik et al., 2019; Zhong et al., 2020; Brikell et al., 2021) is also provided in Table 2.

Compared with the extensive description of the psychiatric comorbidities, the somatic comorbidities of ADHD have received much less attention in the research literature despite the fact that ADHD has been associated with a broad range of medical health problems, such as obesity (Fuemmeler et al., 2011), asthma (Cortese et al., 2018b), migraine (Hansen et al., 2018), type 2 diabetes mellitus, and hypertension (Instanes et al., 2018) and also other somatic diseases. On the other hand, the significant genetic correlations between ADHD and certain somatic traits or diseases (such as weight and weight-related traits, smoking-related cancer, and reproductive traits) reveal a great degree of overlap between the genetic risk factors (Demontis et al., 2019). At this point, it is important to mention that current results support that non-coding variants, such as intronic indels, have been shown to play an important role (Liu et al., 2021) not only in identifying ADHD risk genes (Al-Mubarak et al., 2020), but also for medical diseases (Tan, 2020), as the non-coding elements can regulate the transcription and translation of protein-coding genes. Consistently, recent recommendations for complex, neurodevelopmental disorders increasingly raise the usefulness of whole genome analysis, since the identification of the underlying genetic etiology between neurodevelopmental and somatic conditions can provide a more precise clinical management impacting patient care. This may include initiation of surveillance for disease-related conditions and referrals for further evaluation of associated medical conditions or the possibility to prevent medical comorbidities that may develop later in life (Schaefer and Mendelsohn, 2013; Srivastava et al., 2020; Vanzo et al., 2020). However, there is a need for studies which help to decide whether the same genetic variants influence genetic vulnerability to multiple (psychiatric and somatic) phenotypes (horizontal/independent pleiotropy), or the genetic variants influence vulnerability to one phenotype, and that phenotype in turn causes the other phenotype (Paaby and Rockman, 2013; Verbanck et al., 2018; Vink et al., 2020; Du Rietz et al., 2021a; Zhu, 2021).

Finally, it is important to emphasize that the drugs currently used in ADHD are not curative and thus only allow symptomatic treatment of the disorder. A clearer understanding of the molecular biological background underlying ADHD would be essential for finding causal therapy, to this end, animal models and pluripotent stem cell studies could provide a promising perspective (Tong et al., 2019).

## Discussion

In our brief review, without claiming to be exhaustive, we intended to give an overview of the genetic results relevant to the clinical practice of ADHD and the emerging issues. The clinical need to be addressed by genetic studies can be summarized as the prediction of disease heritability, the identification of diagnostic aids, and the assessment of responsiveness to medication, thus taking into account individual characteristics in the hope of establishing an individualized treatment approach (Zayats and Neale, 2019; Grimm et al., 2020). Table 3 summarizes the potential approaches between research and the clinic. Current genetic results support the trait-based approach, namely that the symptoms of ADHD (similarly to ASD) represent the extremes of a continuous trait that varies in the population. Although the recent genetic results present some new perspectives (e.g., drug-repurposing), they still have many limitations. For example, due to the relative isolation of child and adult studies, we have little bit of knowledge about the genetic background and lifespan perspective of persistence, which is a key issue due to the neurodevelopmental nature of ADHD. The results of the studies with different approaches (candidate gene studies and GWAS) currently show few overlaps, which calls attention to the currently limited knowledge of the genetic architecture of ADHD. These gaps warrant further research and a deeper understanding of the genetic and neurobiological processes underlying ADHD. Moreover, the use of genetic testing in clinical practice should be approached cautiously to avoid the possibility of severe ethical issues.

**TABLE 3:** Summary of potential approaches bridging ADHD genetic research and clinical issues.

Approach	Methods	Results	Limitations	Perspectives	Utility for persisiting ADHD
Candidate-gene association study	Investigation of genetic variants based on a priori neurobiological hypotheses.	Conflicting	Scarcity of longitudinal and normative data. Results based predominantly on patient cohorts.	Future utility for gene-environment interactions, can be connected to neurotransmitter systems, more heuristic for trait-based approaches (e.g. EEG, brain imaging, cognition).	Results do not support this utility.
GWAS/PRS	Investigation of 500.000-1M SNPs without any a priori hypothesis. PRS calculation based on original learning sample and weighted summation of variants.	Promising results, although the interpretation of GWAS hits remains challenging. Can provide information for animal models and cell-based research.	Sample size has to be increased and phenotyping has to be refined, rather than using broad diagnostic categories. Interpretation of PRS scores needs to be extensively studies for clinical samples.	Promising in future for comorbidity and persistence risk-assessment.	Childhood and adult ADHD GWAS data is extensively studies, interpretation remains conflicting.
Cross-disorder analysis	Comparison of GWAS and PRS data across diagnoses.	Results interesting but need further interpretation.	These studies should move from DSM-based diagnostic comparisons to trait-based investigation.	Sheds light on longitudinal course of different diagnostic groups, therefore very interesting for clinicians.	Could provide insight on ADHD comorbidities.
Endophenotypes/Biomarkers	Investigation of neurobiological traits that are more stable than clinical characteristics.	Conflicting	Terminology needs to be improved, causality is often not examined.	Will add to our understanding of underlying neurobiological processes.	Genetic or other biomarker associated with persistence would be extremely important.
Pharmacogenetics	Investigation of genetic variants in receptors and drug-metabolizing enzymes to improve pharmacological treatment.	Is already used for the prediction for metabolism of ADHD medications. Caution is needed for the larger scale implementation of these findings.	Scarcity of data, besides effectiveness, side-effects should also be monitored in such studies.	Drug-repurposing, individual prediction.	Prediction of long-term therapeutic response and side-effects.

The applicability of the current genetic results to the clinical practice is still limited; nevertheless, the focus and the methodology of the studies increasingly accommodate clinical needs. It is also important to highlight that genetic testing seems to be a promising approach for predicting response to treatment and drug tolerability; however, the study results available are still limited. In addition, a more comprehensive determination of the clinical phenotype entailing not only the core symptoms of ADHD but also the related traits and the associated somatic symptoms would prove beneficial for the routine clinical practice. In conclusion, the findings are encouraging for the long-term development of the clinical practice, which is exactly what we need to strive for.

## Author Contributions

LB drafted the first version of the manuscript. JR and AP participated in writing and critical revision of the manuscript. All authors approved the submitted version.

## Conflict of Interest

The authors declare that the research was conducted in the absence of any commercial or financial relationships that could be construed as a potential conflict of interest.

## Publisher’s Note

All claims expressed in this article are solely those of the authors and do not necessarily represent those of their affiliated organizations, or those of the publisher, the editors and the reviewers. Any product that may be evaluated in this article, or claim that may be made by its manufacturer, is not guaranteed or endorsed by the publisher.
